# A segmentation-guided CNN–Vision transformer feature fusion framework for multi-class breast ultrasound image classification

**DOI:** 10.1371/journal.pone.0353628

**Published:** 2026-09-17

**Authors:** Meiru Wu, Jian Wang

**Affiliations:** 1 Department of Ultrasound, The Fifth People’s Hospital of Shanxi Province, Shanxi Provincial Geriatric Hospital, Taiyuan, Shanxi, China; 2 Department of Breast Surgery, Jinan Maternity and Child Care Hospital Affiliated to Shandong First Medical University, Jinan, ShanDong, China; Northeastern University, CHINA

## Abstract

Breast ultrasound (BU) imaging is widely used for detecting breast abnormalities because it is cost-effective, non-invasive, and suitable for dense breast tissue. However, multi-class classification of BU images is considered a challenging task due to low contrast, speckle noise, and overlapping visual patterns between benign and malignant tumours. To address this issue, we develop a segmentation-guided CNN and Vision Transformer based feature fusion framework for efficient multi-class BU image classification. The framework first applies a lesion segmentation model to identify the region of interest by using Fusion-Enhanced Transformer (FET) Unet model. The FET Unet model uses CNNs and Swin Transformers integrated and constructs a UNet-like architecture. In the second step, CNN-based and Vision Transformer-based features are extracted from the segmented lesion regions. In the third step, the CNN and Vision Transformer features are fused to generate a robust feature representation. A deep neural network classifier comprising four dense layers with 1,024, 512, 256, and 128 neurons, respectively, followed by a softmax output layer, is then developed using the fused features to classify breast ultrasound images into benign, malignant, and normal categories. The proposed framework was evaluated using the publicly available Breast Ultrasound Images (BUSI) dataset, which contains 780 ultrasound images collected from women aged 25–75 years, including 437 benign, 210 malignant, and 133 normal cases. Numerical results show that the proposed framework showed 95.11% of accuracy, 95.66% of sensitivity and 97.63% of specificity and F1 score of 0.944644. Based on the classification accuracy, the effectiveness of the proposed hybrid framework is demonstrated in comparison with previously reported methods for multi-class breast ultrasound image classification.

## 1. Introduction

Breast cancer (BC) has been reported to be a rampant and life-threatening diseases among women worldwide [1,2]. Early diagnosis of BD is very crucial as it helps improve survival rates and treatment outcomes. Globally, BC was responsible for approximately 670,000 deaths worldwide in 2022, making it one of the most commonly diagnosed cancers among women [3]. A joint report by the International Agency for Research on Cancer and the WHO published in February 2025 underscores the severity of this public health problem, warning that, by the year 2050, 3.2 million new cases could be diagnosed and 1.1 million deaths could occur [3]. For BC screening and diagnosis, medical imaging techniques such as mammography, magnetic resonance imaging, and ultrasound are widely used. The breast ultrasound (BU) technique has gained significant attention because it is cost-effective, non-invasive, radiation-free, and especially useful for women with dense breast tissue [4]. Due to low contrast, speckle noise, irregular lesion boundaries, and high visual similarity between benign and malignant tumours, BU images are difficult to interpret. As a result, diagnosis often depends on the experience of radiologists, which may introduce subjectivity and variability. For this reason, computer-aided diagnosis systems are becoming increasingly important for improving the accuracy, consistency, and efficiency of breast lesion classification [5,6].

Recently, many machine learning and deep learning based methods have been developed for disease detection and multi class BU image classification containing normal, benign, and malignant ultrasound images [4, 7–9]. Earlier studies mainly used convolutional neural networks and transfer learning models for feature extraction and classification [5,10] or Vision Transformer and hybrid approaches based on CNN and Transformers to improve feature representation [6,11,12]. Other studies used segmentation-guided approaches [13–15]. Despite the recent advancements, low rate of multi-class classification performance is an issue due to class imbalance, noisy image characteristics, and overlapping patterns between benign and malignant lesions [6]. This is evident from the findings of recent studies. For example, Aumente-Maestro et al. proposed an end-to-end prediction approach that were based on refined multi-task framework for BU images. Their approach used deterministic oversampling, and joint segmentation–classification learning with UNet++ and nnU-Net backbones and achieved 0.751–0.754 average Dice score for segmentation and up to 0.802 accuracy with 0.801 weighted F1 for classification. Koshy et al. [16] constructed a hybrid ensemble framework combining EfficientNetB7, DenseNet121, ConvNeXtTiny, XGBoost, and soft-voting strategies for BU image classification. They name their hybrid model HED-Net. Their approach achieved 88.46% accuracy. Rahimnezhad et al. [17] proposed segmentation guided CNN by using U-Net for lesion segmentation and pretrained CNNs i.e. VGG-16, DenseNet-121, DenseNet-169, and ResNet-50 for classification. Their U-Net-DenseNet-169 setting achieved highest accuracy of 87%. Deb and Jha [18] proposed a fuzzy-rank-based ensemble network that combines pretrained VGG-Net, DenseNet, Xception, and Inception models for breast ultrasound image classification. Using five-fold cross-validation on the BU imaging data, their method achieved 85.23% ± 2.52 accuracy. Alotaibi et al. [19] proposed to use a VGG19-based transfer-learning approach and image-fusion preprocessing. The preprocessing included denoising, RGB fusion, and region-of-interest highlighting. Their best results on the BU imaging data was 87.8% classification accuracy. Islam et al. [20] developed an explainable segmentation guided ensemble approach. They used U Net for segmentation, an ensemble of MobileNet and Xception for classification nand Grad-CAM-based explainability for breast ultrasound analysis. On the BU imaging dataset, their model achieved 87.82% testing accuracy and an AUC of 0.91.

Motivated by the above discussed methods and results, to develop an efficient and accurate BU imaging classification approach, this study proposes a segmentation-guided CNN and Vision Transformer-based feature fusion framework for efficient multi-class breast ultrasound image classification. The proposed framework first applies a lesion segmentation model to extract the region of interest from the breast ultrasound image, allowing the classification model to focus mainly on diagnostically meaningful lesion areas. For segmentation, this study uses FET Unet, a hybrid architecture that integrates convolutional neural networks and Swin Transformers within a UNet-like structure [21, 22]. After segmentation, CNN-based features and Vision Transformer-based features are extracted from the lesion-focused images. The CNN features capture local texture, shape, and edge information, while the Vision Transformer features capture global contextual relationships within the lesion region [11,12]. These complementary features are then fused to form a robust and discriminative feature representation. Finally, a deep neural network classifier is developed using the fused features to classify ultrasound images into normal, benign, and malignant classes. The proposed framework achieved an accuracy of 95.11%, sensitivity of 95.66%, specificity of 97.63%, and an F1-score of 0.944644, demonstrating its effectiveness compared with previously proposed methods for multi-class breast ultrasound image classification.

The main contributions of this study are summarized as follows:

- A hybrid segmentation-guided framework is developed that uses FET-UNet to localize lesion regions before classifying breast ultrasound images into normal, benign, and malignant categories.
- In contrast to approaches that rely on either CNN- or ViT-based feature extraction alone, the proposed framework extracts complementary local and global features from lesion-focused images using ResNet50 and ViT-Base, respectively. These features are concatenated to form a fused feature representation.
- The proposed hybrid framework achieved the highest multi-class classification accuracy among the previously reported methods included in the comparative analysis.

## 2. Materials and methods

### 2.1. Dataset description

**Fig 1:**
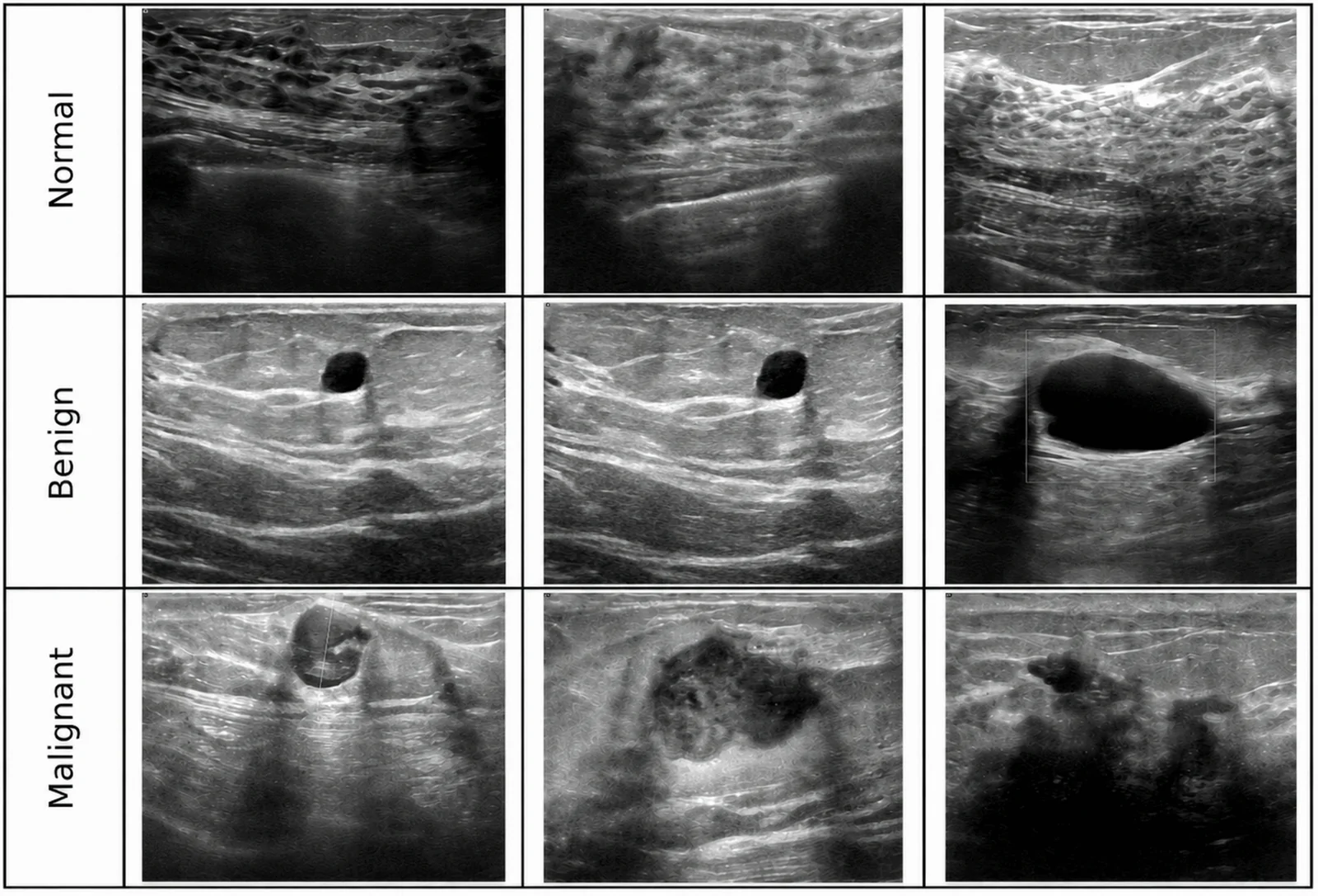
Different samples from different classes of the dataset used in this study.

In this study, we used the Breast Ultrasound Images (BUSI) dataset for multi-class breast ultrasound image classification. The dataset was collected from female patients aged 25–75 years [4]. The dataset contains three diagnostic categories: benign, malignant, and normal. In total, the dataset consists of 780 images, comprising 437 benign cases, 210 malignant cases, and 133 normal cases [4,6]. The images are provided in PNG format with different image resolutions, and binary lesion masks are available for abnormal cases. Different examples belonging to different classes of the dataset are shown in Fig 1. These masks indicate the lesion boundaries annotated by clinical experts and are used by the segmentation models. Since normal images do not contain visible lesion regions, corresponding mask files are not provided for the normal class.

### 2.2. FET-UNet-based segmentation with CNN–vision transformer feature fusion and DNN classification

**Fig 2:**
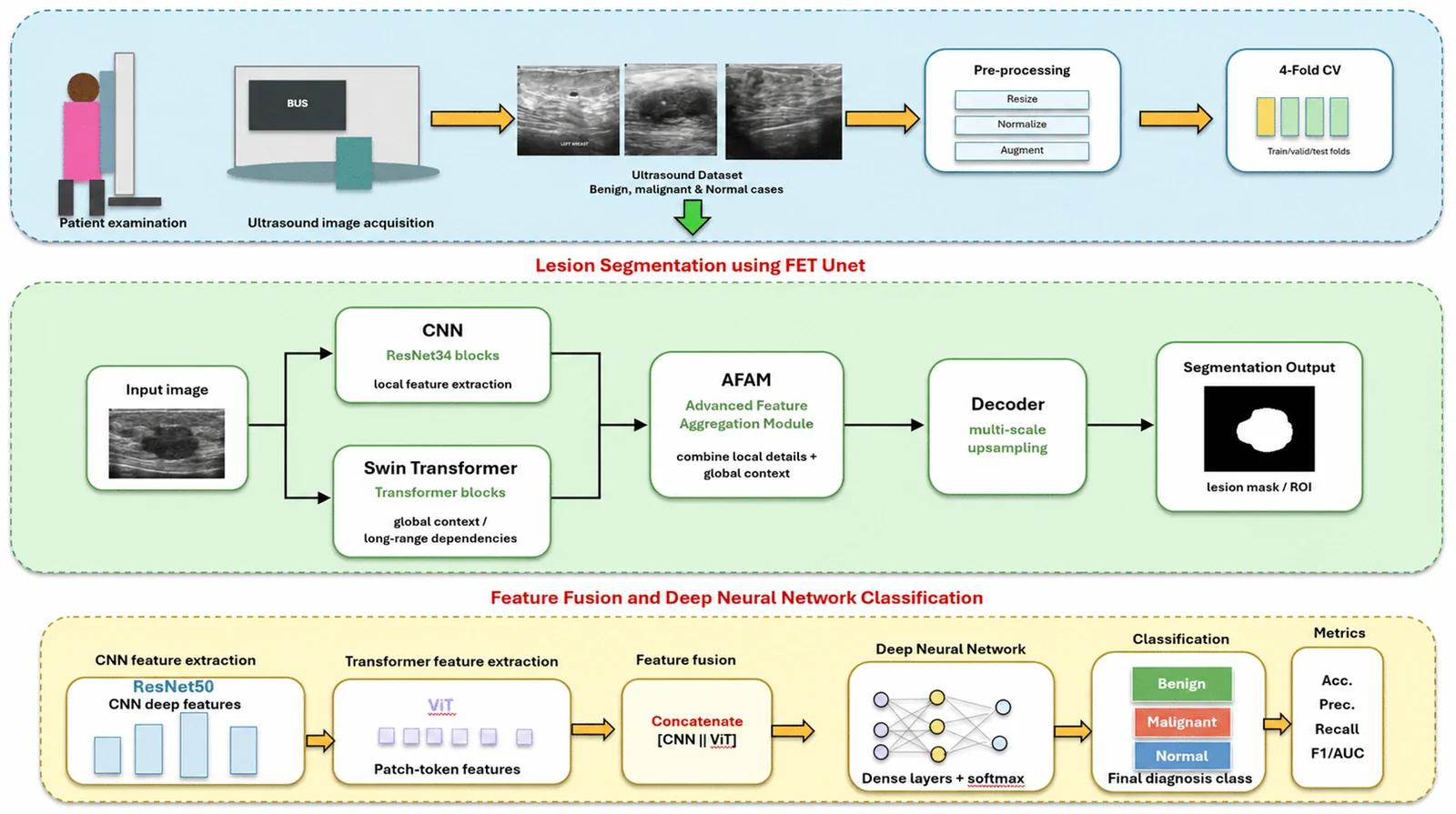
Block diagram showing the working of the proposed framework using FET Unet based segementation and CNN and ViT fused extracted features.

The proposed framework consists of three sequentially connected stages: lesion segmentation, CNN–Vision Transformer feature extraction and fusion, and deep neural network classification and is depicted in Fig 2. The output of each stage serves as the input to the subsequent stage. First, FET-UNet generates a lesion-focused breast ultrasound image. Second, ResNet50 and ViT-Base extract complementary local and global features from the lesion-focused image, which are concatenated to form a fused feature representation. Finally, the fused feature vector is provided to a fully connected deep neural network for classification into normal, benign, and malignant categories.

**Fig 3:**
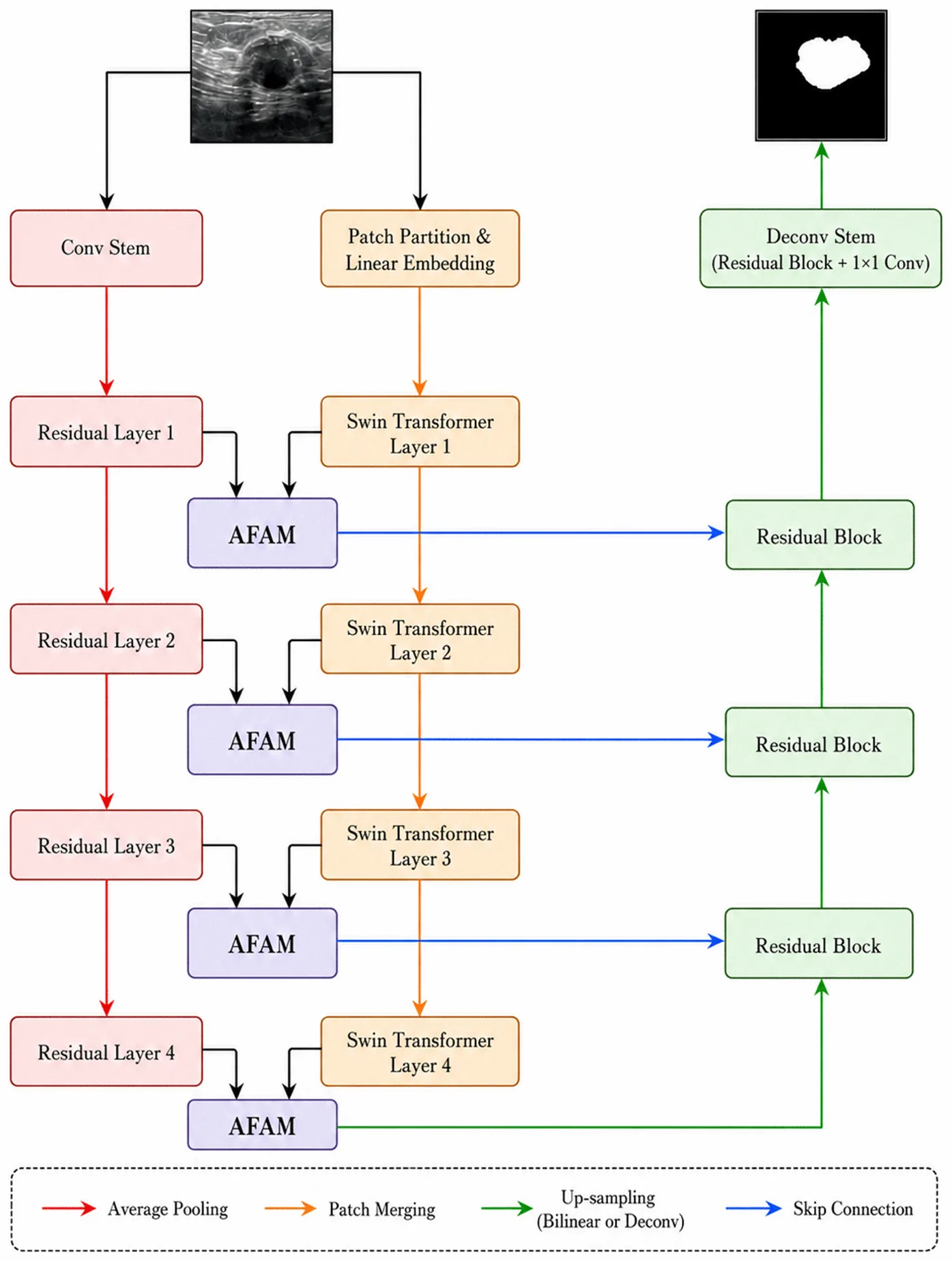
Architecture of the adopted FET-UNet segmentation model.

In the first stage of the proposed framework, FET-UNet was employed as the lesion segmentation model to localise breast tumour regions before feature extraction and classification [21]. FET-UNet is a hybrid encoder–decoder architecture shown in Fig 3, designed to combine the advantages of CNNs and transformer models for effective image segmentation. The main reason for using FET-UNet in this study was to separate the lesion region from the surrounding background tissue so that the subsequent feature extraction and classification stages could use more focused and clinically meaningful image information. This is important because breast ultrasound images usually contain speckle noise, weak contrast, blurred boundaries, and surrounding anatomical structures that may negatively affect classification performance.

The encoder of FET-UNet processes the input image through two parallel feature extraction paths. The first path is based on ResNet-style convolutional blocks, which are effective for learning local texture, boundary, and shape-related features from ultrasound images. The convolutional path was used because lesion margins, internal echo patterns, and small structural variations provide important local information for distinguishing abnormal tissue from background regions. Swin Transformer blocks are used in the second path to divide the image into patches and model relationships between image regions using window-based and shifted-window self-attention [22]. The Swin Transformer path was used to capture broader contextual information and long-range spatial dependencies that may not be fully represented by local convolutional operations. Thus, while the convolutional branch captures fine lesion details, the transformer branch captures the wider spatial context of the ultrasound image.

After feature extraction, FET-UNet uses an Advanced Feature Aggregation Module (AFAM) to combine the outputs of the convolutional and Swin Transformer paths at corresponding encoder stages. In this module, the transformer features are first reshaped to match the spatial structure of the convolutional features. The two feature maps are then combined and passed through a compact convolutional transformation to form a unified representation. Instead of simply joining the features, AFAM further refines the fused representation using attention-based weighting. Spatial attention helps emphasise important lesion-related regions in the image, whereas channel-wise recalibration strengthens the most informative feature channels and suppresses less useful responses. Therefore, AFAM was used to integrate and refine the complementary local and global information obtained from the two encoder paths before lesion-mask reconstruction.

The decoder of FET-UNet gradually reconstructs the segmentation map from the fused encoder features. At each decoding level, the feature map is upsampled and combined with the corresponding encoder information through skip connections. These skip connections help recover spatial information that may be lost during downsampling. Residual blocks are then used to refine the upsampled features and enhance the quality of the predicted lesion mask. Finally, a 1×1 convolution yields the segmentation output, where each pixel is assigned as either lesion or background. In this study, the resulting segmentation mask was used to extract the lesion-focused region of interest from each breast ultrasound image. This segmentation-guided preprocessing reduced the influence of irrelevant background information and provided cleaner inputs for the subsequent CNN and Vision Transformer-based feature extraction, fusion, and deep neural network classification stages. Consequently, the lesion-focused image generated in the first stage directly serves as the input to the second stage of the proposed framework.

After obtaining the lesion-focused images through the FET-UNet segmentation stage, deep feature extraction was performed using two complementary feature extractors: a convolutional neural network and a Vision Transformer. The segmented output image was first resized to 224×224 pixels and converted from grayscale to a three-channel image to make it compatible with the input format of the pre-trained models. For abnormal images, the segmented lesion region was used as the main input for feature extraction. For normal images, where no lesion mask is expected, the resized original ultrasound image was retained when the segmentation model produced an empty mask. This ensured that feature extraction could be performed consistently for all three classes: normal, benign, and malignant.

For CNN-based feature extraction, a pre-trained ResNet50 model was used as the local feature extractor [23]. The final classification layer of ResNet50 was removed, and the remaining network was used only to generate deep image descriptors. ResNet50 was selected because its convolutional operations are effective in capturing local texture, edges, lesion boundaries, shapes, and structural patterns that are important for differentiating benign and malignant breast lesions. The extracted ResNet50 feature vector had a dimensionality of 2,048 for each image.

In parallel, Vision Transformer-based feature extraction was performed using a pre-trained ViT-Base model with a patch size of 16×16 [11]. Unlike CNNs, which process images mainly through local convolutional operations, the Vision Transformer divides the input image into fixed-size patches and learns relationships among these patches through self-attention. ViT-Base was selected to capture global contextual information and long-range spatial dependencies across the lesion-focused image. This information complements the local features extracted by ResNet50 because the appearance of a breast lesion is influenced not only by its local texture but also by its overall shape, spatial distribution, and relationship with surrounding tissue patterns. The ViT model was used as a feature extractor by removing the classification head, producing a 768-dimensional representation for each image.

The CNN and ViT feature vectors were then fused at the feature level using direct concatenation. Let ${𝐟}_{cnn}\in {\mathbb{R}}^{2048}$ represent the feature vector extracted from ResNet50 and ${𝐟}_{vit}\in {\mathbb{R}}^{768}$ represent the feature vector extracted from the ViT-Base model. The final fused feature vector ${𝐟}_{fusion}$ was obtained as:


$$\begin{array}{c} {𝐟}_{fusion}=\left[{𝐟}_{cnn};{𝐟}_{vit}\right], \end{array}$$ (1)

where [;] denotes vector concatenation. Therefore, each breast ultrasound image was represented by a 2,816-dimensional fused feature vector. Direct concatenation was used to preserve the complementary local and global information extracted by ResNet50 and ViT-Base without removing features from either representation. As a result, the fused representation provides a more comprehensive description of the lesion-focused image than either the CNN or Vision Transformer representation alone. The resulting fused feature vector serves as the input to the third stage of the proposed framework.

In the third stage, the 2,816-dimensional fused feature vector is provided as input to a fully connected deep neural network classifier. The classifier comprises four dense layers containing 1,024, 512, 256, and 128 neurons, respectively, followed by a softmax output layer. The DNN was used to learn nonlinear relationships within the combined local and global feature representation and to assign each breast ultrasound image to one of the three classes: normal, benign, or malignant. Thus, the segmentation stage localises the lesion and produces a lesion-focused image, the feature extraction and fusion stage generates a combined representation of local and global image characteristics, and the classification stage uses the fused representation to perform the final multi-class prediction.

## 3. Validation and evaluation of the proposed multiclass classification framework

To assess the proposed multiclass breast ultrasound classification framework, a consistent validation protocol and a set of quantitative performance measures were adopted. For a fair comparison with recent breast ultrasound classification studies, the experimental evaluation followed the validation strategy reported in [21,24]. Specifically, a five-fold cross-validation scheme was employed. The proposed FET-UNet, CNN, and ViT-based classification framework was evaluated using accuracy, sensitivity, specificity, precision, F1-score, and the area under the receiver operating characteristic curve.

In the BUSI dataset, each ultrasound image belongs to one of three categories: benign, malignant, or normal. Let 𝒴={B,M,N} be the set of class labels, where *N*, *B*, and *M* denote normal, benign, and malignant samples, respectively. For each target class y∈𝒴, the multiclass problem is evaluated using a one-versus-all strategy. In this setting, $TP_{y}$ represents the number of samples from class *y* correctly predicted as class *y*, while $FN_{y}$ denotes samples from class *y* that are misclassified as another category. Similarly, $FP_{y}$ indicates samples from the remaining classes that are incorrectly predicted as class *y*, and $TN_{y}$ represents samples from all other classes that are correctly rejected as not belonging to class *y*.

The overall classification accuracy represents the ratio of correctly predicted images to the total number of evaluated images and is computed as


$$\begin{array}{c} \text{Accuracy}=\frac{\sum_{y\in 𝒴}TP_{y}}{\sum_{y\in 𝒴}\left(TP_{y}+FN_{y}\right)}. \end{array}$$ (2)

Sensitivity, also referred to as recall, evaluates the ability of the model to correctly detect samples of a particular class *y*. It is defined as


$$\begin{array}{c} Sen_{y}=\frac{TP_{y}}{TP_{y}+FN_{y}}. \end{array}$$ (3)

Specificity measures how well the model identifies samples that do not belong to class *y* and prevents them from being assigned to that class. It is calculated as


$$\begin{array}{c} Spe_{y}=\frac{TN_{y}}{TN_{y}+FP_{y}}. \end{array}$$ (4)

Precision quantifies the correctness of the samples predicted as class *y*. A higher precision value indicates that fewer samples from other categories are incorrectly assigned to class *y*. It is expressed as


$$\begin{array}{c} Pre_{y}=\frac{TP_{y}}{TP_{y}+FP_{y}}. \end{array}$$ (5)

The F1-score combines precision and sensitivity into a single measure by computing their harmonic mean. For class *y*, it is given as


$$\begin{array}{c} F1_{y}=\frac{2\times Pre_{y}\times Sen_{y}}{Pre_{y}+Sen_{y}}. \end{array}$$ (6)

The same class-wise F1-score can also be expressed directly using the classification counts as follows:


$$\begin{array}{c} F1_{y}=\frac{2TP_{y}}{2TP_{y}+FP_{y}+FN_{y}}. \end{array}$$ (7)

Since the classification task contains three BUSI categories, the class-wise sensitivity, specificity, precision, and F1-score were summarized using macro-averaging. This strategy gives equal weight to each class and is defined as


$$\begin{array}{c} \text{Macro-Sen}=\frac{1}{\left|𝒴\right|}\sum_{y\in 𝒴}Sen_{y}, \end{array}$$ (8)

$$\begin{array}{c} \text{Macro-Spe}=\frac{1}{\left|𝒴\right|}\sum_{y\in 𝒴}Spe_{y}, \end{array}$$ (9)

$$\begin{array}{c} \text{Macro-Pre}=\frac{1}{\left|𝒴\right|}\sum_{y\in 𝒴}Pre_{y}, \end{array}$$ (10)

$$\begin{array}{c} \text{Macro-F1}=\frac{1}{\left|𝒴\right|}\sum_{y\in 𝒴}F1_{y}. \end{array}$$ (11)

For the BUSI dataset, |𝒴|=3. Macro-averaging is suitable for this dataset because the benign, malignant, and normal categories do not contain the same number of images. Therefore, this averaging strategy prevents the majority class from dominating the final evaluation and provides a more balanced assessment of the proposed classification framework.

## 4. Results and discussion

This study presents a segmentation-guided classification framework for multiclass BU image classification. The proposed pipeline first employs FET-UNet for lesion localization, followed by deep feature extraction from the lesion-focused images using convolutional and transformer-based feature extractors. Specifically, ResNet50 and Vision Transformer (ViT) features are extracted and fused to produce a complementary feature representation, which is then used to train a fully connected deep neural network (DNN) for three-class classification of BUSI images into normal, benign, and malignant categories. The hyperparameters of the DNN classifier were optimized by evaluating different candidate configurations. The search space included two, three, and four hidden layers; hidden-layer configurations of [512, 256], [1024, 512, 256], and [1024, 512, 256, 128] neurons; Adam, RMSprop, and stochastic gradient descent optimizers; initial learning rates of 10^–3^, 10^–4^, and 10^–5^; dropout rates ranging from 0.20 to 0.40; and L2 regularization strengths of 10^–3^, 10^–4^, and 10^–5^. Batch sizes of 8, 16, and 32 were also considered. The candidate configurations were evaluated using the same preprocessing procedure and five-fold cross-validation protocol, and the configuration providing the highest and most consistent validation accuracy was selected. All the experiments were performed using Google Colab and Lenovo ThinkPad with Intel(R) Core(TM) Ultra 7 155H (1.40 GHz) processor, and 16.0 GB RAM.

Initially, the FET UNet model was implemented for breast lesion segmentation and compared with several well-established segmentation models. In line with the finding of [21], the FET UNet approach achieved a Dice coefficient of 82.9 ± 2.3%, IoU of 74.7 ± 2.4%, accuracy of 96.8 ± 0.2%, specificity of 98.6 ± 0.4%, and precision of 87.1 ± 3.5%. In comparison, the conventional UNet [25] achieved a Dice score of 70.7 ± 6.1% and IoU of 61.1 ± 3.9%, indicating weaker lesion localization and boundary delineation. Among the CNN-based approaches, R34-DeepLabv3 [26] obtained a Dice score of 80.9 ± 3.2%, IoU of 73.1 ± 2.6%, and accuracy of 96.2 ± 0.6%, while MCRNet [27] achieved the strongest competing performance with a Dice score of 82.2 ± 2.7%, HD95 of 12.4 ± 1.2 mm, IoU of 74.1 ± 2.1%, and accuracy of 96.7 ± 0.5%. Similarly, nnU-Net [28] achieved 81.1 ± 3.1% Dice, 14.8 ± 5.1 mm HD95, and 96.6 ± 0.9% accuracy, whereas AAU-Net [29] obtained 78.5 ± 2.3% Dice and 71.3 ± 2.0% IoU. For the hybrid CNN–transformer models, TransUNet [30], FAT-Net [31], and ATFE-Net [32] achieved Dice scores of 81.0 ± 2.8%, 81.7 ± 2.6%, and 82.1 ± 3.0%, respectively. The transformer-based SwinUnet [33] achieved a Dice score of 79.7 ± 3.3% and IoU of 70.4 ± 3.6%, which was lower than the hybrid CNN–transformer approaches. Overall, FET UNet [21] provided the best overall segmentation performance, surpassing the second-best method, MCRNet, in Dice score, IoU, accuracy, specificity, and precision.

**Fig 4:**
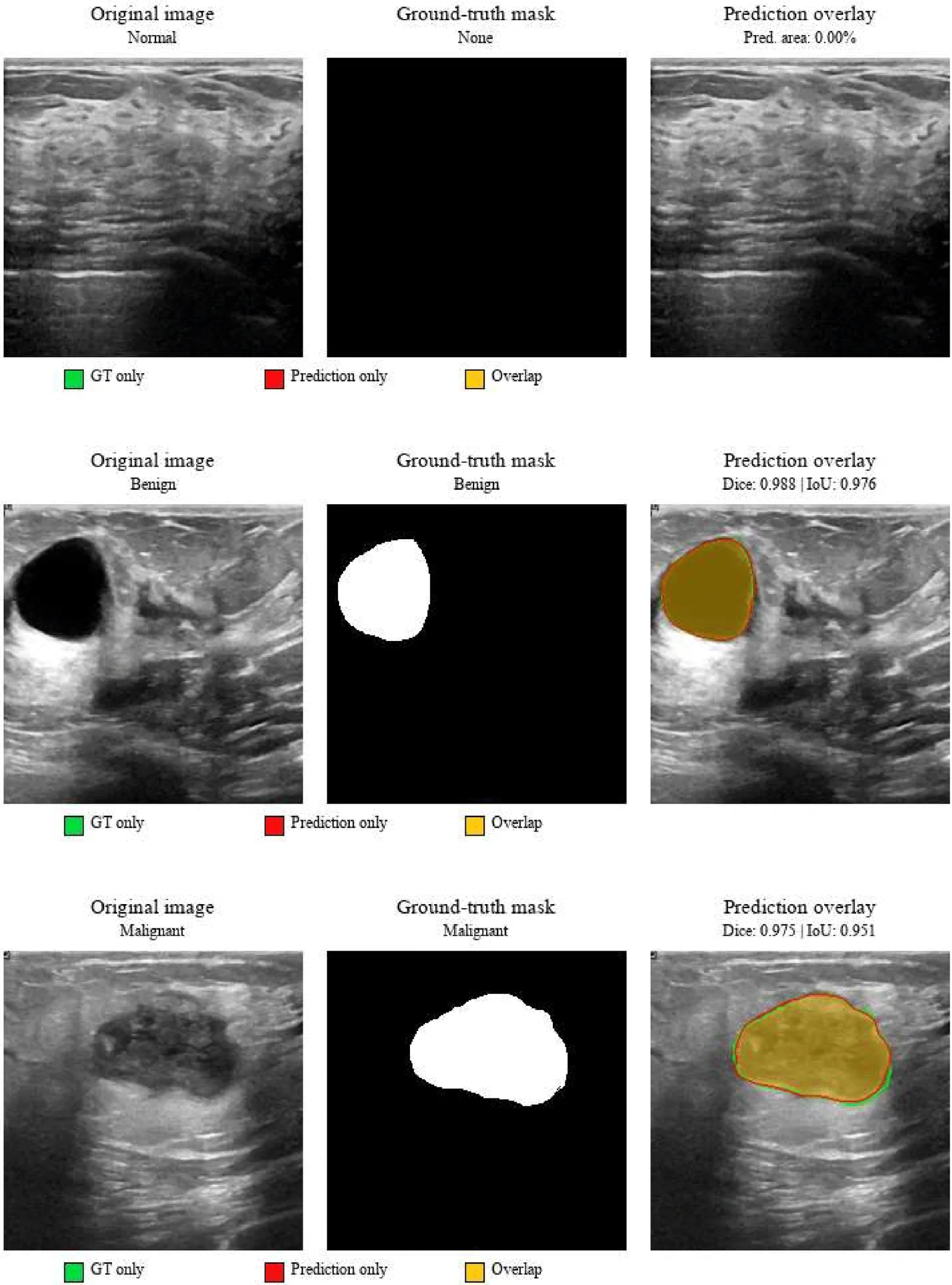
Representative segmentation examples generated using the trained FET UNet models. For each class, the original ultrasound image, ground-truth mask, and prediction overlay are shown. In the overlay, yellow represents correctly segmented overlap, green represents missed ground-truth regions, and red represents extra predicted regions.

Fig 4 shows qualitative segmentation examples obtained using the trained FET UNet model on representative normal, benign, and malignant breast ultrasound images. Each case displays the raw ultrasound image alongside its ground-truth (GT) mask and the corresponding predicted segmentation overlay. In the overlay visualization, yellow indicates the correctly segmented overlap between the predicted mask and GT, green represents lesion regions present in the GT but missed by the model, and red represents additional regions predicted by the model outside the GT. For the normal case, the model correctly produced no lesion region, demonstrating its ability to suppress false-positive segmentation in non-tumor images. For the benign and malignant cases, the predicted masks closely matched the manual annotations, with most of the lesion regions shown in yellow and only minor boundary-level differences. The benign example achieved a Dice score of 0.988 and an IoU of 0.976, while the malignant example achieved a Dice score of 0.975 and an IoU of 0.951. The observations from Fig 4 show that the segmentation model can accurately localize breast lesions across different tumor categories and provide reliable lesion focused regions for subsequent feature extraction.

After lesion localization, deep feature extraction was performed using the FET UNet-guided lesion-focused images. It has been reported that quality of features impacts performance of classification models [34] Thus, for each BUSI image, the predicted mask was applied to the resized ultrasound image to emphasize the lesion region while suppressing irrelevant surrounding background information. In cases where no lesion was predicted, particularly for normal images, the resized original image was retained to preserve useful image-level diagnostic information. Two complementary deep feature extractors were then utilized. The ResNet50 model captured local texture patterns, hierarchical convolutional features, and lesion morphology, whereas the ViT-base patch16–224 branch encoded global contextual information and long-range spatial dependencies. The CNN and ViT feature vectors were then concatenated to form a fused feature vector representation for each image. This produced a final feature matrix of 798 × 2816, corresponding to 133 normal, 454 benign, and 211 malignant BUSI images.

The obtained fused FET UNet-guided CNN–ViT features were given to a fully connected DNN model and its performance was evaluated for three-class classification of normal, benign, and malignant BU images. The DNN model used in this study consisted of four dense layers with 1024, 512, 256, and 128 neurons, respectively, followed by a softmax output layer. Generalization was enhanced and overfitting mitigated through the use of dropout, batch normalization, and L2 regularization, whereas class imbalance across the three categories was handled through class weighting. Under five-fold evaluation, the DNN achieved a mean accuracy of 95.12 ± 8.16%, macro sensitivity of 95.68 ± 7.80%, macro specificity of 97.64 ± 4.11%, and macro F1-score of 94.63 ± 8.79%. Based on the pooled out-of-fold predictions, the DNN achieved an overall accuracy of 95.11% (95% CI: 93.61–96.49%), macro sensitivity of 95.67% (95% CI: 94.08–97.08%), macro specificity of 97.64% (95% CI: 96.86–98.34%), and a macro F1-score of 94.46% (95% CI: 92.69–96.09%). The overall confusion matrix (Fig 8(a)) showed that 129 out of 133 normal images, 428 out of 454 benign images, and 202 out of 211 malignant images were correctly classified, demonstrating the strong discriminative ability of the proposed feature-fusion-based DNN classifier.

**Fig 5:**
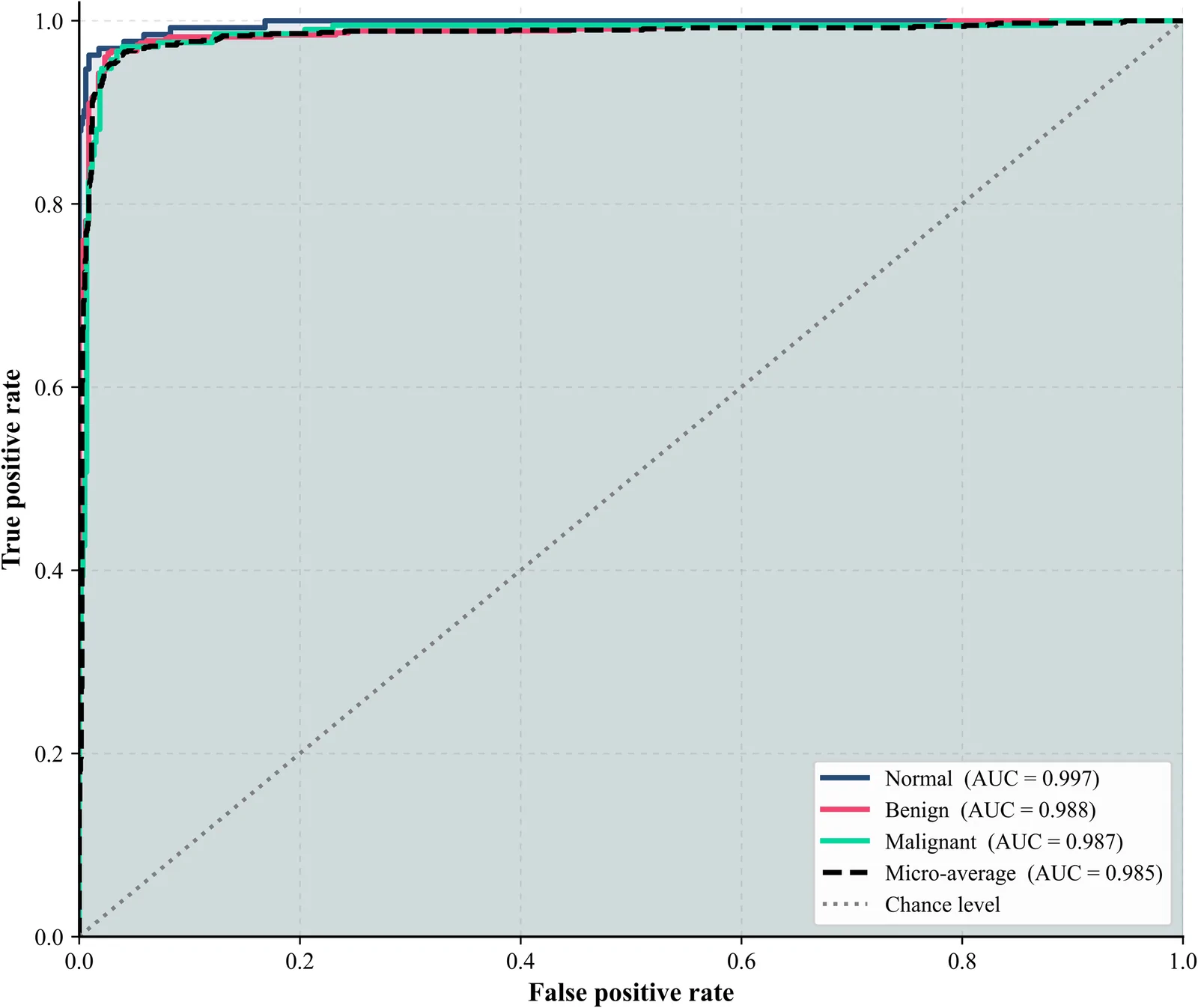
ROC curves of the FET-UNet-guided CNN–ViT feature-fusion-based DNN approach.

**Fig 6:**
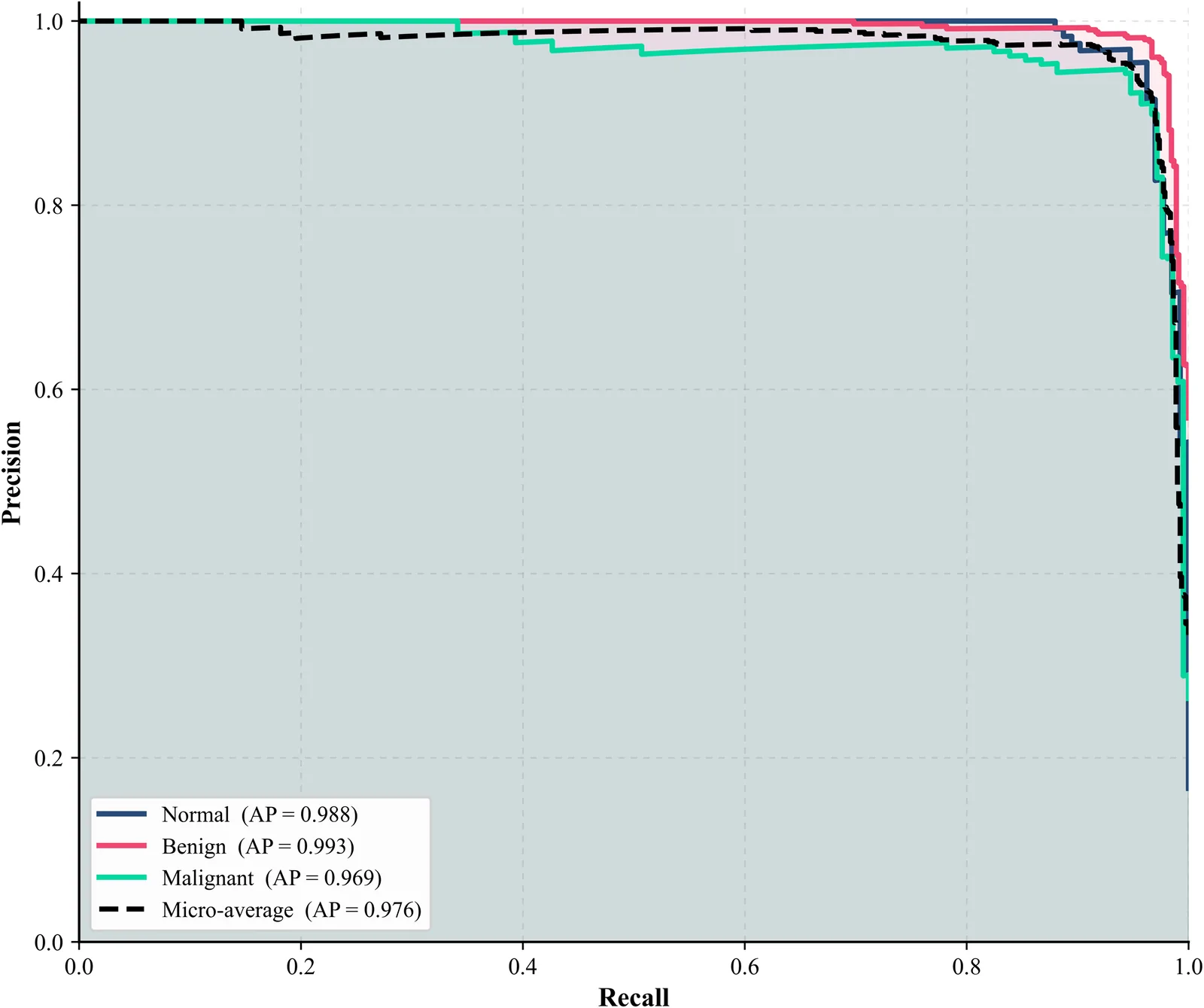
Precision–recall curves of the FET-UNet-guided CNN–ViT feature-fusion-based DNN approach.

**Fig 7:**
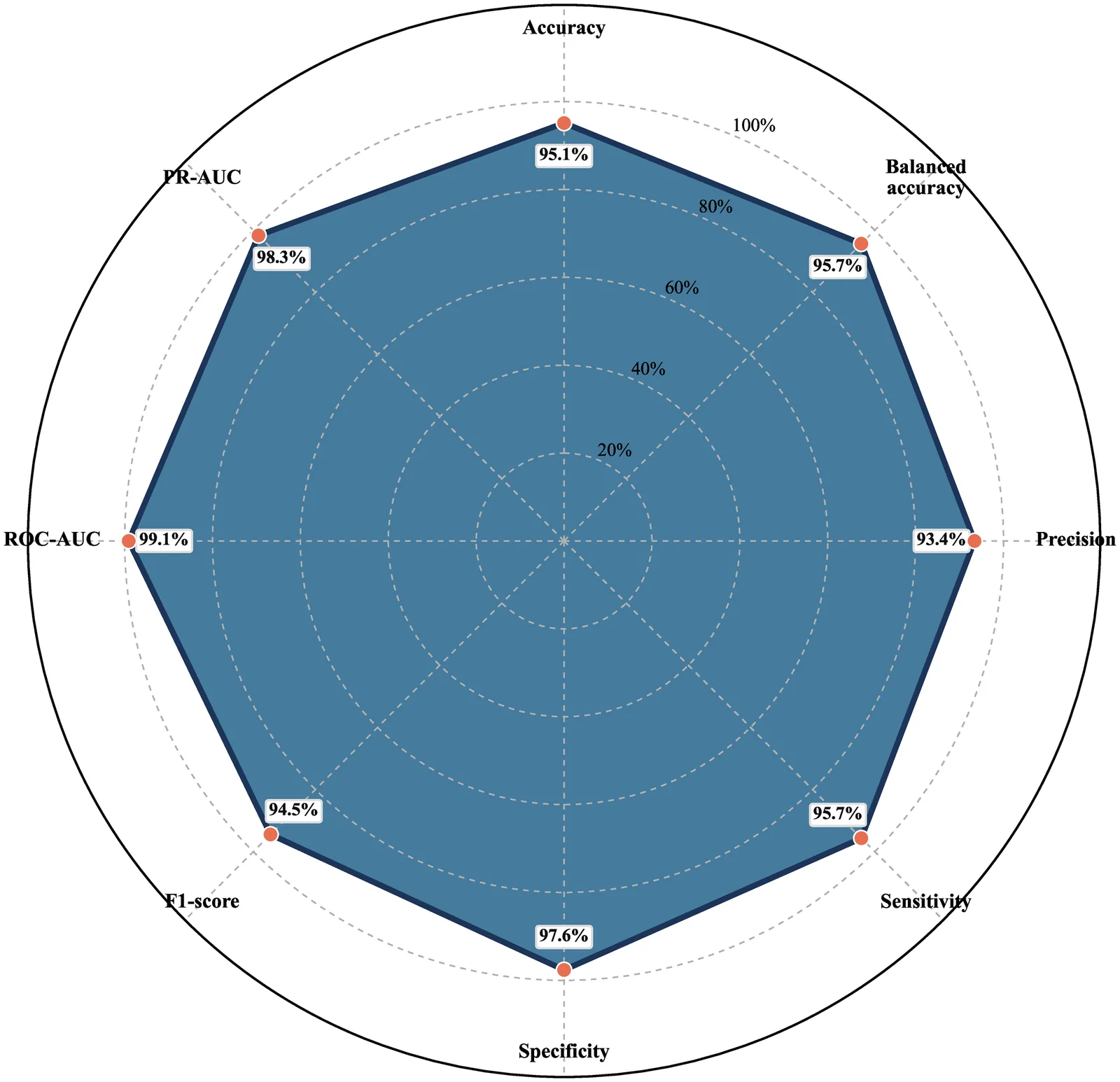
Spider chart summarizing the overall evaluation metrics of the proposed DNN classification approach.

**Fig 8:**
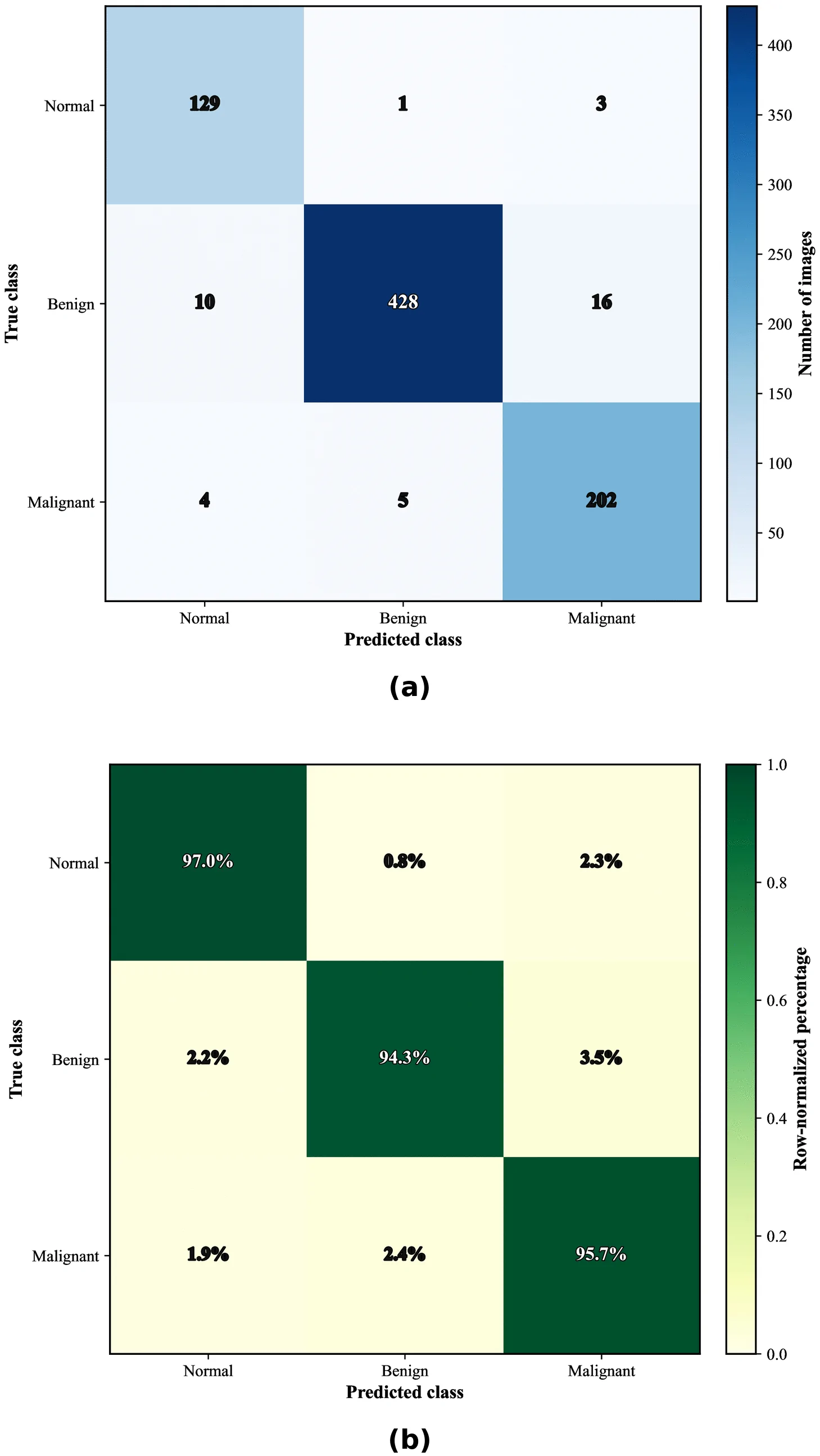
Confusion matrices of the FET-UNet-guided CNN–ViT feature-fusion-based DNN approach. (a) Overall confusion matrix. (b) Normalized confusion matrix.

**Fig 9:**
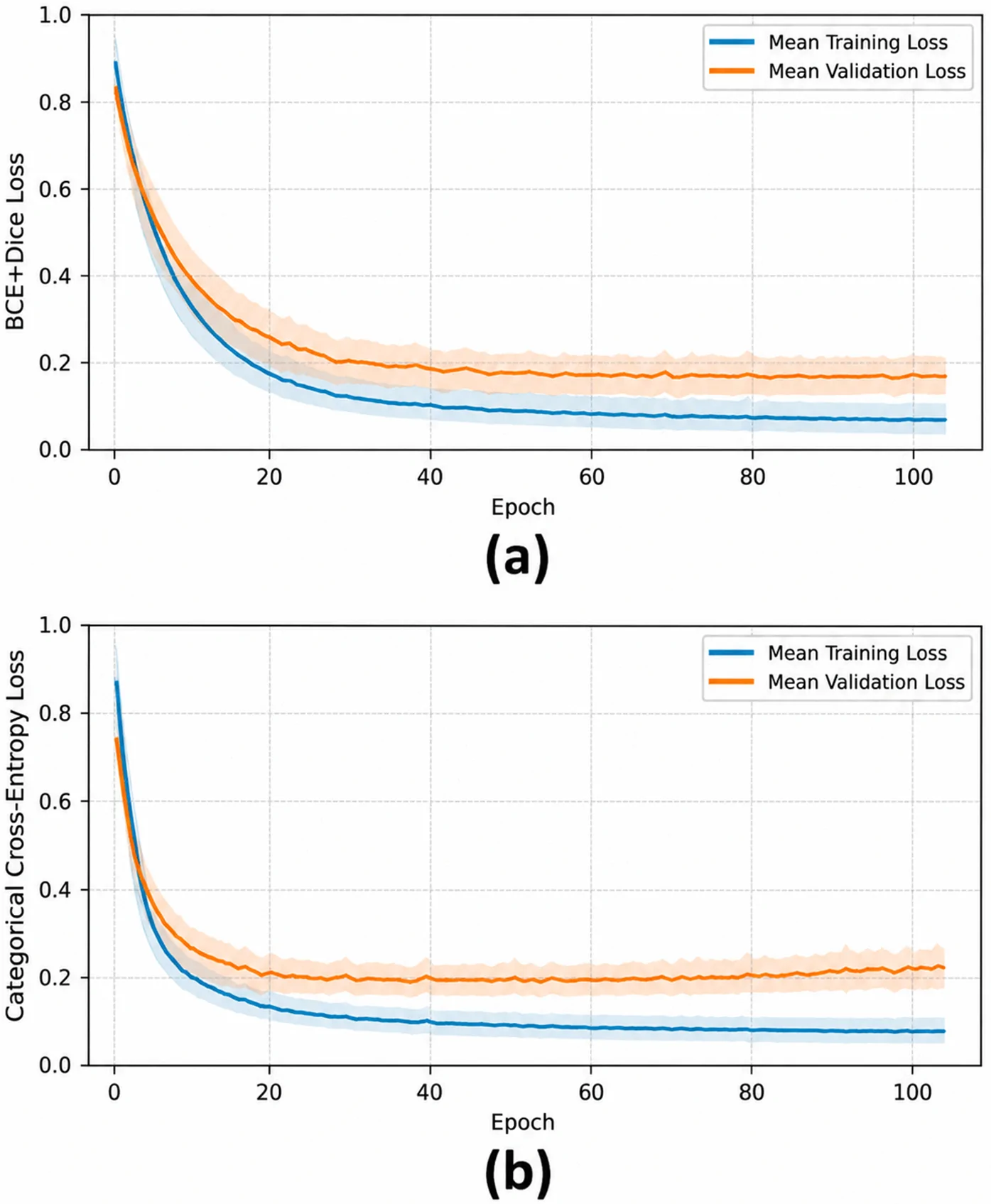
Epoch-wise training and validation loss curves for the trainable components. (a) Epoch-wise training and validation loss curves for the segmentation component. (b) Epoch-wise training and validation loss curves for the classification component.

Figs 5–8 provide a more detailed analysis of the classification performance. The ROC curves in Fig 5 show excellent discriminative capability across the three BUSI classes, with AUC values of 0.997, 0.988, and 0.987 for the normal, benign, and malignant classes, respectively, and a micro-average AUC of 0.985. The precision–recall curves in Fig 6 further confirm the robustness of the model, with average precision values of 0.988 for normal, 0.993 for benign, and 0.969 for malignant cases, along with a micro-average AP of 0.976. These results are particularly important for imbalanced medical datasets, as precision–recall analysis provides a clearer indication of the model’s ability to maintain high positive predictive performance while preserving sensitivity. The spider chart in Fig 7 summarizes the overall behavior of the model across multiple evaluation metrics and shows consistently high values for accuracy, balanced accuracy, precision, sensitivity, specificity, F1-score, ROC-AUC, and PR-AUC. The confusion matrices in Fig 8(b) further demonstrate reliable classification performance, with normalized recognition rates of 97.0%, 94.3%, and 95.7% for normal, benign, and malignant classes, respectively. Additionally, the epoch-wise training and validation loss curves for the trainable components of the proposed framework, i.e., the segmentation component and the deep neural network component, are presented in Fig 9. These results indicate that the proposed two-stage framework benefits from combining segmentation-guided region focusing with hybrid CNN and transformer based feature representation. Most remaining errors occurred between benign and malignant images, which is expected because these two lesion types may exhibit overlapping ultrasound texture, shape, and boundary characteristics. Overall, the results confirm that FET UNet-based lesion localization, when combined with CNN and ViT feature fusion and DNN-based classification, provides a highly discriminative and effective framework for multiclass breast ultrasound image classification.

In addition to evaluating and validating the performance of the proposed framework, we conducted an execution-time analysis of its main components, namely lesion segmentation, feature extraction and fusion, and DNN classification. Using an NVIDIA Tesla T4 GPU in Google Colab, the complete five-fold FET-UNet training and validation process required 4 h 17 min 21 s, comprising 3 h 43 min 27 s for training and 32 min 49 s for validation. The mean training and validation times per fold were 44 min 41 s and 6 min 34 s, respectively. FET-UNet required an average of 19.47 ms to segment a single 224×224 image, corresponding to a throughput of 51.36 images per second. For comparison, under the same cross-validation settings, input resolution, batch size, and number of training epochs, the total training and validation times of conventional U-Net and Swin-Unet were estimated to be approximately 2 h 40 min and 3 h 25 min, respectively. The complete segmentation, ResNet50–ViT feature-extraction, and feature-fusion pipeline processed all images of the dataset in 107.02 s, corresponding to 134.11 ms per image. The complete five-fold DNN classification experiment required 14 min 49 s of time, including 13 min 21 s for training and 1 min 7 s for validation.

Table 1 compares the computational complexity and parameter counts of conventional U-Net, Swin-Unet, and the implemented FET-UNet. The computational cost of conventional U-Net is dominated by convolutional operations and can be expressed as $O\;\left(\sum_{l=1}^{L_{c}}N_{l}K_{l}^{2}C_{l-1}C_{l}\right)$, where $N_{l}=H_{l}W_{l}$ denotes the number of spatial positions at layer *l*, $K_{l}$ denotes the convolutional kernel size, and $C_{l-1}$ and $C_{l}$ denote the input and output channe*l* dimensions, respectively.

**Table 1:** Computational complexity and parameter counts of the FET Unet, Conventional Unet and Swin Unet segmentation models.

Model	Computational Complexity	Parameters (M)
Conventional U-Net	$O\;\left(\sum_{l=1}^{L_{c}}N_{l}K_{l}^{2}C_{l-1}C_{l}\right)\backslash )$	≈31.04
Swin-Unet	$O\;\left(\sum_{l=1}^{L_{s}}B_{l}N_{l}\left(C_{l}^{2}+M^{2}C_{l}\right)\right)\backslash )$	≈27.15
FET-UNet	$O\;\left(\sum_{l\in 𝒞}N_{l}K_{l}^{2}C_{l-1}C_{l}+\sum_{l\in 𝒮}B_{l}N_{l}\left(C_{l}^{2}+M^{2}C_{l}\right)+\sum_{l\in 𝒜}N_{l}\left(C_{l}^{R}+C_{l}^{S}\right)C_{l}^{F}\right)\backslash )$	53.27

^a^$N_{l}=H_{l}W_{l}$ denotes the number of spatial positions or tokens at stage *l*; $K_{l}$ is the convolutiona*l* kernel size; $C_{l-1}$ and $C_{l}$ are the input and output channel dimensions; $B_{l}$ is the number of Swin Transformer blocks at stage *l*; *M* is the fixed attention-window size; and $C_{l}^{R}$, $C_{l}^{S}$, and $C_{l}^{F}$ denote the ResNet, Swin Transformer, and fused AFAM channel dimensions, respectively. The sets 𝒞, 𝒮, and 𝒜 denote the convolutional, Swin Transformer, and AFAM stages. The expressions report the dominant computational terms. Parameter counts depend on the precise model configuration.

For Swin-Unet, the dominant computational complexity can be expressed as $O\;\left(\sum_{l=1}^{L_{s}}B_{l}N_{l}(C_{l}^{2}+M^{2}C_{l})\right)$, where $B_{l}$ denotes the number of Swin Transformer blocks at stage *l* and *M* denotes the fixed attention-window size. Because self-attention is restricted to local windows, the computationa*l* complexity scales approximately linearly with the number of image tokens when the window size and channel dimensions are fixed.

The computational complexity of the FET-UNet includes the convolutional operations of the ResNet34 encoder and residual decoder, the shifted-window attention operations of the Tiny Swin Transformer branch, and the AFAM-based feature-fusion operations. Its dominant complexity can therefore be expressed as $O\;\left(\sum_{l\in 𝒞}N_{l}K_{l}^{2}C_{l-1}C_{l}+\sum_{l\in 𝒮}B_{l}N_{l}(C_{l}^{2}+M^{2}C_{l})+\sum_{l\in 𝒜}N_{l}(C_{l}^{R}+C_{l}^{S})C_{l}^{F}\right)$, where $C_{l}^{R}$, $C_{l}^{S}$, and $C_{l}^{F}$ denote the ResNet, Swin Transformer, and fused AFAM channel dimensions, respectively.

The FET-UNet contained 53.27 million parameters, whereas commonly used standard configurations of conventional U-Net and Swin-Unet contain approximately 31.04 million and 27.15 million parameters, respectively. The larger parameter count of FET-UNet results from incorporating parallel convolutional and Transformer-based feature-extraction branches, AFAM-based multi-scale feature fusion, and a residual convolutional decoder. The parameter counts of conventional U-Net and Swin-Unet may vary slightly depending on their implementation-specific channel configurations and output layers.

We further conducted a stage-wise failure-case analysis to examine the limitations of the proposed framework. Classification errors are summarized by the confusion matrices presented in Fig 8. For the segmentation stage, each benign and malignant image was evaluated using the FET-UNet model corresponding to the fold in which that image was included in the validation set. Therefore, the analysis was based on out-of-fold predictions from models that had not been trained on the evaluated images. The mean Dice scores were 0.8277 ± 0.2160 for benign lesions and 0.7587 ± 0.2158 for malignant lesions, while the corresponding mean IoU scores were 0.7478 ± 0.2302 and 0.6492 ± 0.2232, respectively. These results indicate that malignant lesions were more difficult to segment than benign lesions.

**Fig 10:**
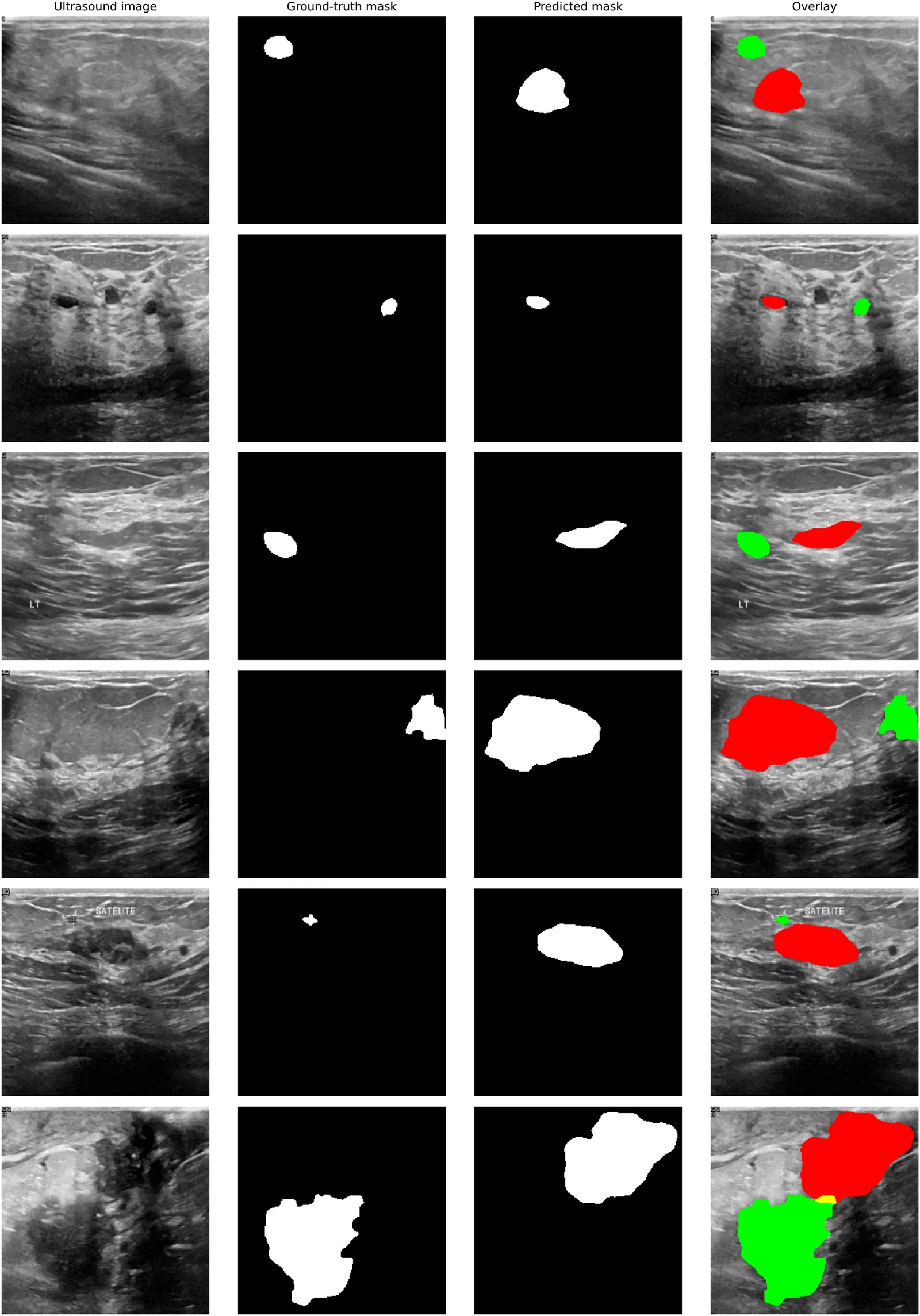
Failure case analysis of the segmentation stage.

The three lowest-Dice cases from each lesion class were selected for visual examination. As shown in Fig 10, the principal failure patterns were over-segmentation and spatial localization or boundary mismatch. In several cases, the predicted mask was located away from the annotated lesion, resulting in little or no overlap between the predicted and ground-truth regions.

For the feature extraction and fusion stage, the fused representations were analysed using a five-fold centroid-based procedure. Within each fold, feature standardization and the normal, benign, and malignant class centroids were calculated using only the training samples. Each validation representation was then compared with the three training-fold centroids. A case was considered difficult when its nearest centroid belonged to a class different from its true class.

**Fig 11:**
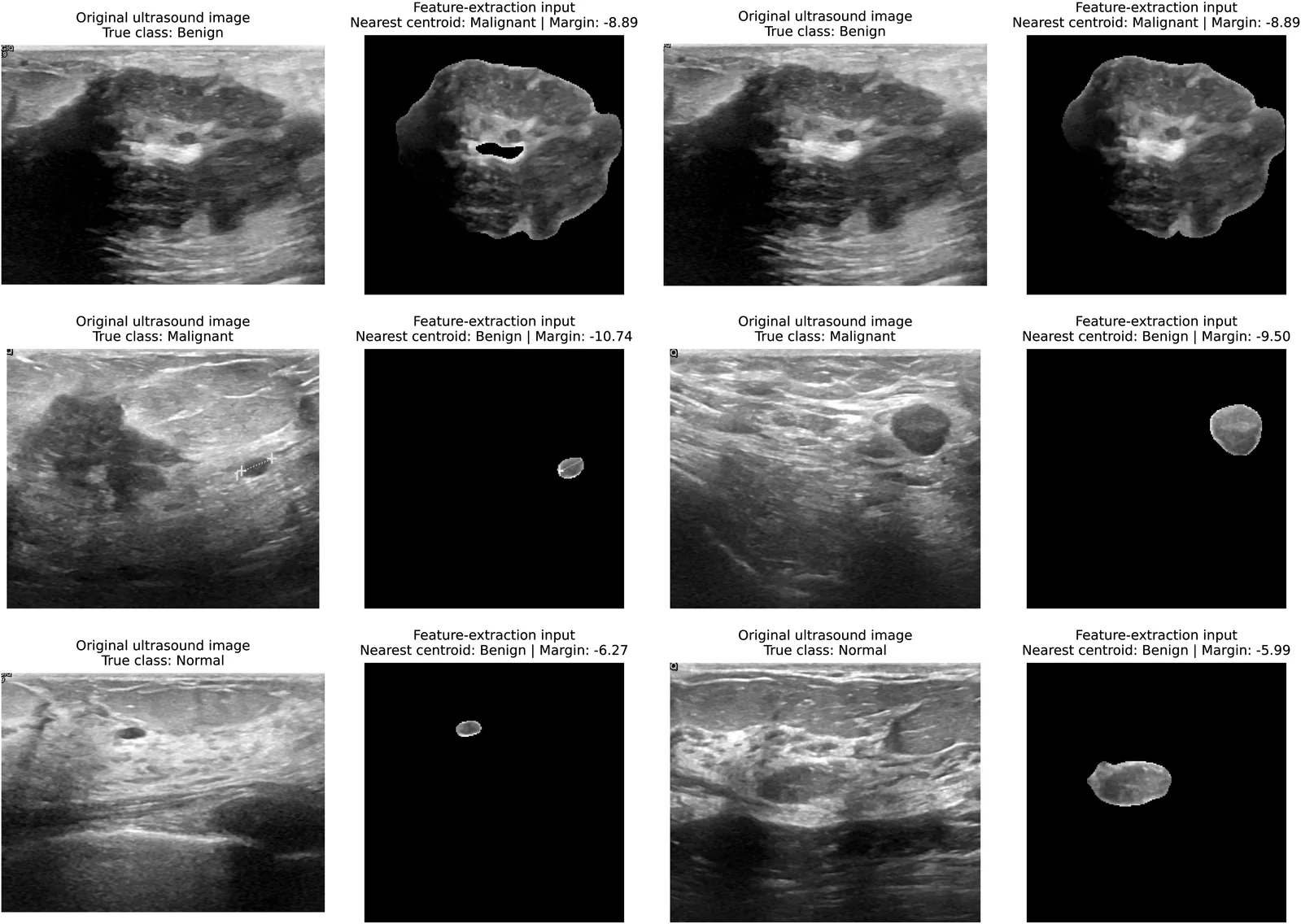
Failure case analysis of the feature-extraction and fusion-stage.

27.95% of the total fused feature representations were closer to another class centroid than to their true-class centroid. This included 121 of 437 benign cases (27.69%), 64 of 210 malignant cases (30.48%), and 33 of 133 normal cases (24.81%). The highest rate of ambiguous representations was observed for the malignant class. Representative difficult cases are shown in Fig 11. The selected benign cases were closer to the malignant centroid, whereas the selected malignant and normal cases were closer to the benign centroid. These findings demonstrate that some lesion-focused images produce overlapping local and global feature representations, reducing the separation among the three classes.

An ablation study was designed based on component- and stage-wise replacement or removal, to estimate the contribution of the principal stages of the proposed framework. All the experiments of ablation study were conducted and evaluated using the same cross-validation strategy, preprocessing procedure, and experimental settings. In the first two experiments, for the segmentation-stage comparison, the proposed FET-UNet was replaced with either a conventional CNN-based U-Net or a Transformer-based Swin-Unet, while the subsequent ResNet50 and ViT-Base feature-fusion and DNN classification stages were kept unchanged. The conventional U-Net configuration achieved an accuracy of 88.97%, whereas the Swin-Unet configuration achieved 90.48%. To evaluate the individual contributions of the feature-extraction branches, the FET-UNet outputs were processed using CNN model, i.e., CNN (ResNet50) alone or ViT-Base alone. The CNN-only configuration achieved an accuracy of 93.36%, whereas the ViT-Base-only configuration achieved 91.98%. The contribution of the proposed DNN classifier was further examined by replacing it with a multiclass support vector machine while retaining the same FET-UNet segmentation and fused ResNet50–ViT-Base features. The multiclass SVM configuration achieved an accuracy of 87.59%. Overall, the complete framework, comprising FET-UNet-based lesion segmentation, ResNet50 and ViT-Base feature extraction, feature concatenation, and DNN-based classification, provided the highest classification accuracy among the evaluated configurations. All these results are reported in Table 2.

**Table 2:** Performance of different methods of the ablation study.

Variant	Segmentation	Feature Extraction	Classifier	Accuracy (%)
E1	Conventional U-Net	ResNet50 + ViT-Base	DNN	88.97
E2	Swin-Unet	ResNet50 + ViT-Base	DNN	90.48
E3	FET-UNet	ResNet50 only	DNN	93.36
E4	FET-UNet	ViT-Base only	DNN	91.98
E5	FET-UNet	ResNet50 + ViT-Base	Multiclass SVM	87.59
E6	FET-UNet	ResNet50 + ViT-Base	DNN	95.11

To validate the effectiveness of the proposed FET Unet, CNN and ViT based approach, we compared its results with recently published methods. This is summarized in the Table 3.

**Table 3:** Comparative classification performance of the proposed FET Unet and CNN ViT based framework with the existing studies.

Study	Method	Accuracy (%)	F1-score (%)	AUC
Al-Dhabyani et al. [10] (2019)	GAN + CNN	94.00 ± 0.40	–	–
Moon et al. [35] (2020)	CNN-based image fusion	90.77 ± 0.49	82.86 ± 0.49	0.949
Xu et al. [36] (2022)	MTL-COSA	91.48 ± 0.57	93.59 ± 0.62	0.970
Xu et al. [37] (2023)	RMTL-Net	91.02 ± 0.62	93.32 ± 0.62	0.967
He et al. [24] (2024)	Multitask segmentation and classification	94.44 ± 2.07	–	–
Wen et al. [38] (2025)	DenseNet121	–	0.85	0.93
Jiang et al. [39] (2026)	YOLOv7 and YOLOv8 transfer learning	89.4	–	0.830
Paçal [5] (2022)	Vision Transformer	88.6	–	–
Gheflati and Rivaz [12] (2022)	ResNet	83.0	–	–
Ali et al. [40] (2023)	Ensemble Meta-Model	90.0	–	–
Xiong et al. [41] (2023)	UNet + ResNet + SIFT	88.6	–	–
Deb and Jha [18] (2023)	fuzzy-rank-based ensemble of CNNs	85.23 ± 2.52		
Alotaibi et al. [19] (2023)	VGG19 + image-fusion	87.8		
Islam et al. [20] (2024)	explainable segmentation guided ensemble approach.	87.82		0.91
Wei et al. [15] (2024)	MFFMT	95.0	–	–
Montaha et al. [42] (2024)	Graph Convolutional Network	91.03	–	–
Işık and Paçal [43] (2024)	Prototypical Networks and MAML	88.9	–	–
Aumente-Maestro et al. [44] (2025)	80.2	0.801		
Rahimnezhad et al. [17] (2025)	U-Net + DenseNet-169	87		
Saini et al. [45] (2025)	CNN	93.0	–	–
Yıldırım et al. [6] (2025)	ROI + ViT + MLP + Ensemble + Focal Loss	94.03	–	–
Koshy et al. [16] (2026)	HED-Net	88.46		

## 5. Conclusion and future work

This study proposed a segmentation-guided CNN and Vision Transformer feature fusion framework for multi-class breast ultrasound image classification. The main objective was to address the low rate of classification performance and to improve the classification of normal, benign, and malignant breast ultrasound images by first localizing the lesion region and then extracting more informative and complementary deep features from the focused region of interest. For lesion localization, FET UNet was used because of its ability to combine convolutional feature learning with transformer-based contextual modelling. The segmentation stage helped reduce the influence of irrelevant background tissue, speckle noise, and surrounding anatomical structures, thereby providing cleaner and more diagnostically meaningful inputs for the classification stage. After segmentation, ResNet50 and ViT-based feature extractors were used to capture complementary information from the lesion-focused ultrasound images. The ResNet50 branch extracted local texture, boundary, and structural features, while the ViT branch captured global contextual relationships and long-range spatial dependencies. These features were fused to form a more robust feature representation and were then used to develop a deep neural network classifier. The proposed framework achieved strong classification performance, with an overall accuracy of 95.11%, macro sensitivity of 95.67%, macro specificity of 97.64%, and macro F1-score of 94.46%. The ROC and precision-recall analyses further confirmed the robustness of the proposed approach, with high class-wise AUC and average precision values across normal, benign, and malignant categories.

Although the proposed framework demonstrated strong performance under cross-validation on the benchmark BUSI dataset, its generalizability to independently collected datasets remains unverified. Differences in ultrasound acquisition devices, imaging protocols, image quality, patient populations, class distributions, and annotation procedures may introduce domain shifts and affect its performance on external data. Therefore, future studies should evaluate the framework on independent breast ultrasound datasets containing comparable diagnostic categories and investigate the effects of domain-related differences on classification performance. If substantial domain-related performance degradation is observed, domain-adaptation and domain-generalization strategies, such as domain-adversarial training, maximum mean discrepancy-based feature alignment, correlation alignment, self-supervised pretraining, and test-time adaptation, could be incorporated into the framework to improve its robustness and generalizability across independently collected datasets.
